# Osmophobia and allodynia are critical factors for suicidality in patients with migraine

**DOI:** 10.1186/s10194-015-0529-1

**Published:** 2015-05-12

**Authors:** Sung-Pa Park, Jong-Geun Seo, Won-Kee Lee

**Affiliations:** Department of Neurology, School of Medicine, Kyungpook National University, 680 Gukchaebosang-ro, Jung-gu, Daegu, 700-842 Republic of Korea; Center of Biostatics, School of Medicine, Kyungpook National University, Daegu, Republic of Korea

**Keywords:** Suicide, Osmophobia, Allodynia, Migraine, Risk factor, Depression, Anxiety

## Abstract

**Background:**

Sensory hypersensitivities are common phenomena in migraine. We examined the role of sensory hypersensitivities on suicidality in patients with migraine.

**Methods:**

Patients with migraine (with or without aura) were consecutively recruited from our headache clinic. We asked them if they experienced photophobia, phonophobia, osmophobia, and allodynia during migraine attack. The Mini International Neuropsychiatric Interview was used to diagnose current major depressive disorder (MDD), current generalized anxiety disorder (GAD) and suicidality.

**Results:**

Among 220 subjects, 25.5 % had current MDD, 17.3 % had current GAD, and 31.8 % had suicidality. Patients with suicidality were more like to have a low household income, chronic migraine (CM), medication overuse headache, high headache intensity, osmophobia, allodynia, high disability, MDD, and GAD than those without suicidality. The strongest risk factor for suicidality by multivariate analyses was osmophobia (adjusted Odds Ratio [AOR] 3.12, 95 % confidence interval [CI] 1.57-6.21, *p* = 0.001), followed by current MDD (AOR 2.99, 95 % CI 1.33-6.76, *p* = 0.008), CM (AOR 2.48, 95 % CI 1.21-5.09, *p* = 0.013), current GAD (AOR 3.11, 95 % CI 1.22-7.91, *p* = 0.017), and allodynia (AOR 2.72, 95 % CI 1.19-6.21, *p* = 0.018).

**Conclusions:**

Osmophobia and allodynia are critical factors for suicidality in patients with migraine, after controlling for depression, anxiety, and CM.

## Background

Adolescent and adult patients with migraine have been known to have a high risk of suicidality including suicidal ideation and attempt [1–6]. Risk factors for developing suicide in patients with migraine were psychiatric disorders [1–6], female gender [2], migraine with aura [1–3], higher headache frequency [3] and pain intensity [4, 6], younger age [5], unmarried state [5], and higher disability [5]. Among them, migraine with aura has been chosen as a migraine-specific factor for suicide after controlling for age, gender, major depression, and anxiety disorders [1–3].

Sensory hypersensitivities such as photophobia, phonophobia, osmophobia, and allodynia were frequently observed in patients with migraine [7–12]. Although the frequency of allodynia is widely variable (15.1 % to 69.7 %) [10–12], the frequencies of photophobia, phonophobia, and osmophobia were in the range from 43 % to 86 % [7–9, 11, 12]. Recently, a hospital-based study in Taiwan reported that patients with osmophobia were more likely to have higher level of depression and anxiety than those without osmophobia [7]. In addition, patients with allodynia had a higher frequency of anxiety thacn those without allodynia and those with moderate to severe allodynia had a higher risk of developing depression than those with no or mild allodynia [11].

Despite the significance of migraine with aura for suicidality, relevant studies did not consider sensory hypersensitivities as risk factors of suicide despite the existence of the relationship between sensory hypersensitivities and psychiatric symptoms [7, 11]. Therefore, we investigated whether sensory hypersensitivities contributed to suicidality in patients with migraine.

## Methods

### Subjects

We invited new patients with migraine who had consecutively visited a headache clinic in the Department of Neurology at Kyungpook National University Hospital since September 2013. Patients were adolescents and adults between the ages of 15 and 75. They were newly diagnosed patients at the visit or already diagnosed patients but did not take preventive medicines for migraine and other psychotropic agents within a month. A diagnosis of migraine was assigned based on the International Classification of Headache Disorders, 3rd edition, beta version (ICHD-3 beta) by a trained neurologist (SP Park) [13]. Patients who had illiteracy, mental retardation, serious medical, neurological, or psychiatric disorders, and alcohol or drug abuse that prevented them from cooperating in the psychiatric interview and understanding the questionnaire were excluded. Patients who had a probable migraine or who declined to participate in the interview were also excluded.

### Study design

A cross-sectional study was conducted as part of a hospital-based study which examined the impact of psychiatric disorders on migraine. The Institutional Review Board of Kyungpook National University Hospital approved the study. All participants obtained written informed consent. SP Park interviewed each patient and reviewed the patient’s medical charts to collect demographic and clinical information for a computerized database. Sociodemographic data were collected on variables including age, gender, education, place of residence (city or country), religion, employment, household income (earning at least three million KRW per month, equivalent to 2800 USD per month or not), and marital status (married or unmarried, divorced, and bereaved). Clinical data included; type of migraine, migraine chronicity (episodic migraine [EM] or chronic migraine [CM]), medication overuse headache (MOH), age at onset, disease duration, headache intensity, accompanying symptoms (presence of nausea and/or vomiting, photophobia, phonophobia, osmophobia, or allodynia) and family history. The Visual Analog Scale (VAS) measured headache intensity. The VAS was measured in two different ways, i.e. the VAS_max_ and VAS_now_. VAS_max_ meant the maximal intensity of headaches experienced during the prior three months, and VAS_now_ represented the intensity of the headache on the day of the psychiatric interviews. Photophobia, phonophobia, and osmophobia were defined as the hypersensitivity to light, sound, and certain odors during migraine attacks which could cause avoidance of those stimulations or aggravation of migraine symptoms. We asked patients whether they experienced symptoms in the preceding year. Allodynia was measured using the 12-item Allodynia Symptom Checklist (ASC-12) [14] with a cut-off score of >2 defining allodynic patients. Family history of migraine was defined as the existence of migraine diagnosis in a lineal ascendant and siblings. Migraine disability was measured by the Migraine Disability Assessment Scale (MIDAS) [15]. The overall level of disability was represented as follows; grade I, little or no disability (score of 0–5), grade II, mild disability (6–10), grade III, moderate disability (11–20), and grade IV, severe disability (21 or more). Disability of grade I and II was compared to that of grade III and IV.

### Psychiatric interviews

All participants were interviewed by a neuropsychologist within a week after the first visit to determine whether they had current major depressive disorder (MDD), current generalized anxiety disorder (GAD) or suicidality using the Korean version of the Mini-International Neuropsychiatric Interview-Plus 5.0.0. (MINI) [16]. The MINI is a brief, structured interview based on DSM-IV criteria [17], and is recommended to screen for psychiatric comorbidity in headache patients [18]. The Kappa values of MDD and GAD in the Korean version were 0.71 and 0.57, respectively, which exhibited good agreement between the MINI and the expert’s diagnosis. The suicidality module is composed of six questions of different weight, including five questions asking about current suicidality (which includes suicidal ideation or attempt in the preceding month: wish for death [weight 1], wish for self-harm [weight 2], suicidal thought [weight 6], suicide plan [weight 10], and suicide attempt [weight 10], and one question asking the lifetime suicide attempt [weight 4]). If respondents said “yes” for at least one of the six questions, they were thought to have suicidality. The degree of current suicidality is estimated from the sum of the weighted score of the six questions, with low (1–5), moderate (6–9), and high (≥10) level of suicide risk.

### Statistical analyses

The Statistical Package for the Social Sciences (SPSS version 19.0) was used for the analyses of data. Descriptive statistics are presented in terms of counts, percentages, means, and standard deviations. To determine factors for suicidality, a logistic regression model with suicidality as the outcome was developed, using a two-step procedure. First, univariate logistic regression analyses were used to test which potential variables were associated with suicidality among patients with migraine. Second, all variables with a statistically significant univariate effect were entered in a multivariate logistic regression model and a forward likelihood ratio test was chosen to assess the differential contribution of individual variables to suicidality. Results were reported as odds ratio (OR) with 95 % confidence intervals, standardized regression coefficients (Beta) and corresponding *p* values. The level of statistical significance was set at *p* < 0.05.

## Results

A total of 254 patients with migraine consecutively visited our headache clinic. Among them, 34 patients were excluded because of refusal to interview (*n* = 12), probable migraine (n = 10), taking preventive medicine within a month (*n* = 4), age older than 75 (*n* = 4), mental retardation (*n* = 2), or illiteracy (*n* = 2). Subsequently, 220 patients (30 men/190 women, mean age 40.1 ± 13.1) completed the study. Among them, 17 patients (7.7 %) had migraine with aura and 203 patients (92.3 %) had migraine without aura. EM was manifested in 95 patients (43.2 %), CM in 125 patients (56.8 %), and MOH in 52 patients (23.6 %). All patients with MOH had CM. Photophobia, phonophobia, osmophobia, and allodynia were manifested in 104 patients (47.3 %), 140 patients (63.6 %), 108 patients (49.1 %), and 39 patients (17.7 %), respectively. Current MDD and current GAD were revealed in 56 patients (25.5 %) and 38 patients (17.3 %), respectively. Suicidality was documented in 70 patients (31.8 %). Among them, 25 patients (35.7 %) had a moderate to high degree of suicidality. Current suicidality, lifetime suicide attempt, or both were revealed in 38 patients (54.3 %), 18 patients (25.7 %), and 14 patients (20 %), respectively. Forty patients (57.1 %) had only suicidal ideation and 30 patients (42.9 %) had current or lifetime suicidal attempt.

Sociodemographic, clinical, and psychiatric aspects of migraine with respect to suicidality are listed in Table 1. Factors contributing to suicidality by univariate analyses are documented in Table 2. Patients with suicidality were more like to have a low household income (OR 1.88, 95 % confidence interval [CI] 1.04-3.41, *p* = 0.038), CM (OR 3.05, 95 % CI 1.63-5.69, *p* < 0.001), MOH (OR 2.27, 95 % CI 1.20-4.32, *p* = 0.012), high VAS_max_ score (OR 0.80, 95 % CI 0.68-0.94, *p* = 0.007), high VAS_now_ score (OR 0.81, 95 % CI 0.72-0.91, *p* < 0.001), osmophobia (OR 2.98, 95 % CI 1.64-5.41, *p* < 0.001), allodynia (OR 4.70, 95 % CI 2.27-9.71, *p* < 0.001), high MIDAS grade (OR 3.05, 95 % CI 1.63-5.69, *p* < 0.001), current MDD (OR 5.50, 95 % CI 2.87-10.54, *p* < 0.001), and current GAD (OR 5.86, 95 % CI 2.77-12.40, *p* < 0.001) than those without suicidality. Age, gender, education, and the type of migraine were not associated with suicidality. The frequency of osmophobia or allodynia was not significantly changed by the degree of suicidality or if patients had only suicidal ideation or attempted suicide. In addition, it was not affected by if patients had current suicidality or lifetime suicidal attempts.

**Table 1:** Sociodemographic, clinical, and psychiatric aspects of eligible subjects (*n* =220) with respect to suicidality

	Mean ± SD (range) or number (%)	
	Suicidality	No suicidality
Characteristic	(*n* = 70)	(*n* = 150)
Age, years	40.3 ± 13.2 (16–73)	40.0 ± 13.0 (15–65)
Gender, female	62 (88.6)	128 (85.3)
Education, years	12.4 ± 2.9 (6–18)	13.0 ± 2.8 (5–18)
Place of residence, city	61 (87.1)	125 (83.3)
Religion, yes	36 (51.4)	81 (54.0)
Employment, yes	34 (48.6)	72 (48.0)
Household income, at least 3 million KRW/month	41 (58.6)	109 (72.7)
Marital status, no history of divorce or bereavement	42 (60.0)	95 (63.3)
Type of migraine		
Migraine with aura	4 (5.7)	13 (8.7)
Migraine without aura	66 (94.3)	137 (91.3)
Migraine chronicity		
Episodic migraine	18 (25.7)	77 (51.3)
Chronic migraine	52 (74.3)	73 (48.7)
Family history of migraine	45 (64.3)	96 (64.0)
MOH	24 (34.3)	28 (18.7)
Age of onset	29.7 ± 12.0 (9–53)	30.5 ± 12.3 (8–59)
Disease duration	10.6 ± 9.4 (0.3-36)	9.4 ± 7.3 (0.2-37)
VAS_max_^a^	8.5 ± 1.5 (3–10)	7.7 ± 2.2 (0–10)
VAS_now_^b^	3.2 ± 2.6 (0–10)	1.9 ± 2.3 (0–8)
Accompaning symptoms		
Nausea and/or vomiting	60 (85.7)	132 (88.0)
Photophobia	33 (47.1)	71 (47.3)
Phonophobia	51 (72.9)	89 (59.3)
Osmophobia	47 (67.1)	61 (40.7)
Allodynia	24 (34.3)	15 (10.0)
MIDAS score	42.0 ± 41.4 (0–190)	18.3 ± 21.9 (0–135)
MIDAS grade		
Grade I and II	18 (25.7)	77 (51.3)
Grade III and IV	52 (74.3)	73 (48.7)
Current MDD	34 (48.6)	22 (14.7)
Current GAD	25 (35.7)	13 (8.7)

Abbreviations: KRW = Korean Won; MOH = medication overuse headache; VAS = Visual Analog Scale, MIDAS = Migraine Disability Assessment Scale; MDD = major depressive disorder; GAD = generalized anxiety disorder

^a^Maximal headache intensity during the preceding 3 months

^b^Headache intensity on the day of psychiatric interviews

**Table 2:** Factors contributing to suicidality by univariate analyses

Variable	*β*	SE (*β*)	OR (95 % CI)	*p* value
Age	−0.002	0.011	1.00 (0.98-1.02)	0.862
Gender	0.287	0.441	1.33 (0.56-3.16)	0.516
Education	0.076	0.051	1.08 (0.98-1.19)	0.134
Place of residence	0.304	0.419	1.36 (0.60-3.08)	0.468
Religion	−0.103	0.29	0.90 (0.51-1.59)	0.722
Employment	0.023	0.29	1.02 (0.58-1.81)	0.937
Household income	0.631	0.304	1.88 (1.04-3.41)	0.038
Marital status	0.141	0.297	1.15 (0.64-2.06)	0.635
Type of migraine	−0.448	0.591	0.64 (0.20-2.03)	0.448
CM	1.114	0.319	3.05 (1.63-5.69)	<0.001
Family history of migraine	0.012	0.302	1.01 (0.56-1.83)	0.967
MOH	0.821	0.328	2.27 (1.20-4.32)	0.012
Age at onset	0.006	0.012	1.01 (0.98-1.03)	0.640
Disease duration	−0.018	0.018	0.98 (0.95-1.02)	0.304
VAS_max_^a^	−0.221	0.082	0.80 (0.68-0.94)	0.007
VAS_now_^b^	−0.208	0.059	0.81 (0.72-0.91)	<0.001
Nausea and/or vomiting	−0.201	0.424	0.82 (0.36-1.88)	0.636
Photophobia	−0.008	0.290	0.99 (0.56-1.75)	0.979
Phonophobia	0.610	0.316	1.84 (0.99-3.42)	0.054
Osmophobia	1.092	0.304	2.98 (1.64-5.41)	<0.001
Allodynia	1.547	0.371	4.70 (2.27-9.71)	<0.001
MIDAS grade	1.114	0.319	3.05 (1.63-5.69)	<0.001
Current MDD	1.704	0.332	5.50 (2.87-10.54)	<0.001
Current GAD	1.767	0.383	5.86 (2.77-12.40)	<0.001

Abbreviations: CM = chronic migraine; MOH = medication overuse headache; VAS = Visual Analog Scale, MIDAS = Migraine Disability Assessment Scale; MDD = major depressive disorder; GAD = generalized anxiety disorder

^a^Maximal headache intensity during the preceding 3 months

^b^Headache intensity on the day of psychiatric interviews

Risk factors contributing to suicidality by multivariate analyses are documented in Table 3. The strongest risk factor for suicidality was osmophobia (Beta 0.314, adjusted OR [AOR] 3.12, 95 % CI 1.57-6.21, *p* = 0.001), followed by current MDD (Beta 0.264, AOR 2.99, 95 % CI 1.33-6.76, *p* = 0.008), CM (Beta 0.249, AOR 2.48, 95 % CI 1.21-5.09, *p* = 0.013), current GAD (Beta, 0.237, AOR 3.11, 95 % CI 1.22-7.91, *p* = 0.017), and allodynia (Beta 0.211, AOR 2.72, 95 % CI 1.19-6.21, *p* = 0.018). A forward likelihood ratio test produced a five-variable model that explained 34.3 % of the variance in suicidality. According to Beta, the contribution of osmophobia to suicidality was 1.19 times greater than that of current MDD, 1.26 times greater than that of CM, 1.32 times greater than that of current GAD, and 1.49 times greater than that of allodynia.

**Table 3:** Factors contributing to suicidality by multivariate analyses

Variable	*β*	SE (*β*)	Beta^a^	Adjusted OR (95 % CI)	*p* value
Constant	−2.577	0.547		0.08	<0.001
Osmophobia	1.138	0.351	0.314	3.12 (1.57-6.21)	0.001
Current MDD	1.096	0.416	0.264	2.99 (1.33-6.76)	0.008
CM	0.909	0.367	0.249	2.48 (1.21-5.09)	0.013
Current GAD	1.134	0.476	0.237	3.11 (1.22-7.91)	0.017
Allodynia	1.000	0.422	0.211	2.72 (1.19-6.21)	0.018

Abbreviations: MDD = major depressive disorder; CM = chronic migraine; GAD = generalized anxiety disorder

^a^Standardized regression coefficients

## Discussion

Sensory hypersensitivities such as photophobia and phonophobia were frequently observed in patients with migraine and included in the diagnostic criteria of migraine of the International Classification of Headache Disorders (ICHD)-3 beta [13]. The other types of hypersensitivities, osmophobia and allodynia, were also frequently manifested in patients with migraine [7–12], but were not included in the diagnostic criteria of migraine. Although osmophobia has been reported as one of the specific symptoms for differentiating migraine from other headache disorders [7–9], it remains under-recognized as a migraine-associated symptom, and its clinical implications are under-investigated [7]. Under these circumstances, we found that osmophobia and allodynia were major risk factors for suicidality in patients with migraine, after controlling for MDD, GAD, and CM.

Regarding risk factors for suicidality in patients with migraine, several studies reported psychiatric disorders, sociodemographic factors, type of migraine, headache frequency and intensity, and migraine disability [1–6]. We also found similar results in univariate analyses. However, sensory hypersensitivities, such as osmophobia and allodynia, were found to be critical factors by multivariate analyses. We also found that the contribution of osmophobia to suicidality is a little higher and the contribution of allodynia to suicidality is a little lower than that of depression or anxiety. This means that osmophobia and allodynia are important to determine suicidality as much as psychiatric disorders.

The mechanism to explain the contribution of osmophobia to suicidality is unknown. One possibility is a common pathway of osmophobia and mood changes may provoke suicidality. Smell is innately related to the limbic system. In an event-related fMRI study, olfactory stimulation during migraine attack produced increased limbic and brainstem activity [19]. In mood disorders, several limbic areas have been reported to be altered in structural, functional and molecular neuroimaging [20]. Therefore, altered function of the limbic system appears to produce psychiatric disorders as well as osmophobia, and finally can provoke suicidality. However, this theory may be insufficient to explain the mechanism in our patients, because osmophobia can contribute to suicidality regardless of the psychiatric disorders. Another possibility is osmophobia is associated with headache intensity, and severe pain accompanying osmophobia may provoke suicidality. In a population-based study, headache severity at baseline predicted suicide attempts during the two-year follow-up period in migraine or non-migraine headache [4]. In a hospital-based study, headache intensity was a risk factor for suicidal ideation in patients with migraine after controlling for depression [6]. Through these results, if osmophobia is an expression for severe pain, it can be a risk factor for suicidality. Further studies are needed to elucidate these relationships. The Mechanism for the impact of allodynia to suicidality is a little easier to explain. Patients with allodynia can reveal suicidality not only by a high frequency of comorbid depression and anxiety [11, 12] but also by a high probability of migraine chronification [10]. Of course, allodynia may also be an expression for severe pain. The relationship of them should be clarified.

Our study failed to detect that migraine with aura was an important risk factor for suicidality. There may be several reasons to explain such. First, relevant studies were population-based studies with a large sample sizes [1–3]. On the other hand, our study was a hospital-based study, and the number of patients who had migraine with aura was 17. Therefore, the selection bias may affect the result. Second, previous studies were conducted in adolescents (12–15 years old) [2, 3] or young adults (21–30 years old) [1] with migraine, but not in patients of all ages. We investigated risk factors of suicidality in patients who were 15 to 73 years old (mean 40.1 years old). Therefore, the different age groups of eligible patients can elicit different results. In a large sample, community-based study, migraine with aura was not a risk factor for suicidal attempt in patients who were 25 to 55 years old (mean 40.4 years old) [4]. A further study to investigate risk factors according to the different age groups should be needed. Third, they did not consider the impact of sensory hypersensitivities on suicidality [1–3] despite the existence of a relationship between osmophobia or allodynia and psychiatric symptoms [7, 11]. Fourth, they did not investigate the impact of CM in relation to episodic migraine (EM) on suicide [1–3]. As expected, CM results in substantially greater disability than EM. In a hospital-based study in Korea, CM sufferers were more likely to have depression, anxiety, disability, and impaired quality of life than EM sufferers [12]. In a review of the literatures, authors summarized that patients with chronic daily headache such as CM and chronic tension-type headache appears to have a high suicidal intent due to depression, disability, impaired quality of life and chronic pain [21]. Therefore, CM should be included as a variable to determine suicide. After all, if we investigate risk factors for suicidality in patients with migraine considering these issues, more precise data will be obtained.

Our study has some limitations. First, this is a hospital-based study, which may not represent the whole migraine population. Therefore, a large sample, population-based study should be done. Second, the cross-sectional nature of our study prevented us from drawing conclusions about the causal relationship between osmophobia or allodynia and suicidality. A longitudinal study is needed. Third, ethnic differences could also limit the generalization of our results, for example, our patients revealed a higher frequency of osmophobia and a lower frequency of allodynia than Western patients did. Further studies to prove our results should also be conducted in Western countries. Fourth, we did not measure olfactory function during the ictal period of the migraine. Subjective feelings and objective findings of olfaction may draw different conclusions. Fifth, we did not investigate the impact of comorbid organic diseases on suicidality. However, we excluded patients who had serious medical, neurological, or psychiatric disorders from the study, so the impact of other organic diseases on suicidality is likely to be trivial.

## Conclusions

Suicide is the tenth leading cause of death worldwide [22] with about 800,000 to one million people dying annually, giving a mortality rate of 11.6 per 100,000 persons per year [23]. In a two year population-based observational study, suicidal attempt is 4.43 times higher in patients with migraine than in healthy controls when sex, psychiatric disorder, and previous history of suicidal attempts had been adjusted for at baseline [4]. Almost 32 % of our patients manifested suicidality and nearly 43 % of them attempted suicide when they visited a tertiary care hospital. Under these circumstances, clinicians should always observe carefully the existence of suicidality in patients with migraine who visit outpatient clinics. In a busy clinical setting, psychiatric interviews, including suicidality, take a long time to conduct. In this situation, simply asking the patients about osmophobia or allodynia will be an initial step for further evaluation of suicidality. A global study to evaluate sensory hypersensitivities as a red flag for suicidality should be done in the future.

## Abbreviations

ICHD-3: International Classification of Headache Disorders
EM: Episodic migraine
CM: Chronic migraine
MOH: Medication overuse headache
VAS: Visual Analog Scale
ASC: Allodynia Symptom Checklist
MIDAS: Migraine Disability Assessment Scale
MDD: Major depressive disorder
GAD: Generalized anxiety disorder
MINI: Mini-International Neuropsychiatric Interview
OR: Odd Ratio
